# High-Resolution Melting-Curve Analysis of *obg* Gene to Differentiate the Temperature-Sensitive *Mycoplasma synoviae* Vaccine Strain MS-H from Non-Temperature-Sensitive Strains

**DOI:** 10.1371/journal.pone.0092215

**Published:** 2014-03-18

**Authors:** Muhammad A. Shahid, Philip F. Markham, Marc S. Marenda, Rebecca Agnew-Crumpton, Amir H. Noormohammadi

**Affiliations:** 1 Faculty of Veterinary Science, The University of Melbourne, Werribee, Victoria, Australia; 2 Asia-Pacific Centre for Animal Health, Faculty of Veterinary Science, The University of Melbourne, Victoria, Australia; Miami University, United States of America

## Abstract

Temperature-sensitive (*ts* ^+^) vaccine strain MS-H is the only live attenuated *M. synoviae* vaccine commercially available for use in poultry. With increasing use of this vaccine to control *M. synoviae* infections, differentiation of MS-H from field *M. synoviae* strains and from rarely occurring non-temperature-sensitive (*ts* ^–^) MS-H revertants has become important, especially in countries where local strains are indistinguishable from MS-H by sequence analysis of variable lipoprotein haemagglutinin (*vlhA*) gene. Single nucleotide polymorphisms (SNPs) in the *obg* of MS-H have been found to associate with *ts* phenotype. In this study, four PCRs followed by high-resolution melting (HRM)-curve analysis of the regions encompassing these SNPs were developed and evaluated for their potential to differentiate MS-H from 36 *M. synoviae* strains/isolates. The nested-obg PCR-HRM differentiated *ts* ^+^ MS-H vaccine not only from field *M. synoviae* strains/isolates but also from *ts* ^–^ MS-H revertants. The mean genotype confidence percentages, 96.9±3.4 and 8.8±11.2 for *ts* ^+^ and *ts* ^–^ strains, respectively, demonstrated high differentiating power of the nested-obg PCR-HRM. Using a combination of nested-obg and obg-F3R3 PCR-HRM, 97% of the isolates/strains were typed according to their *ts* phenotype with all MS-H isolates typed as MS-H. A set of respiratory swabs from MS-H vaccinated specific pathogen free chickens and *M. synoviae* infected commercial chicken flocks were tested using obg PCR-HRM system and results were consistent with those of *vlhA* genotyping. The PCR-HRM system developed in this study, proved to be a rapid and reliable tool using pure *M. synoviae* cultures as well as direct clinical specimens.

## Introduction

*Mycoplasma synoviae* causes airsacculitis and infectious synovitis in chickens and turkeys [1]. It causes significant economic losses to the poultry industry due to carcass condemnation, culling of lame birds and deterioration in eggshell quality [2], [3]. The temperature-sensitive (*ts* ^+^) strain MS-H (Vaxsafe MS®, Bioproperties Pty. Ltd. Australia) is the only live attenuated vaccine available and is used in several countries to control *M. synoviae* infections in poultry flocks.

Differentiation of MS-H from field strains is an important step to establish whether a flock is free from wild-type *M. synoviae*. It is also important to establish whether the vaccine strain has colonised the respiratory mucosa so as to produce an efficient immune response to protect against wild-type disease. A number of PCR-based techniques have been reported for typing of *M. synoviae* strains, targeting the *vlhA* gene [4]-[7], 16S rRNA genes [8] or the 16S to 23S rRNA intergenic spacer region [9], [10]. Only a small number of these studies included the MS-H vaccine in their experiments. Jeffery et al. [11] described a combination of PCR and high-resolution melting (HRM) curve analysis of the *vlhA* gene products to discriminate a large number of *M. synoviae* strains, although their system did not differentiate MS-H from several Australian field strains as they shared the same *vlhA* gene sequence. The *vlhA*-based typing system however should be useful in other countries as MS-H like strains are believed to be rare, if not absent, outside Australia. Pulsed-field gel electrophoresis (PFGE) using *Bln*I and *Bam*HI digestions coupled with *vlhA* gene sequencing was useful in differentiating the MS-H from Japanese *M. synoviae* strains/isolates [12]. Also a PCR based cycling probe technology (CPT) developed by Ogino et al. [13], targeting an A→G substitution at 365^th^ nucleotide from the 5′ conserved region of *vlhA* gene, has been claimed useful for MS-H differentiation from Japanese *M. synoviae* strains/isolates. However techniques reported in both of these reports are time consuming and may be difficult to perform on a routine basis in diagnostic laboratories. More importantly, none of the techniques reported above have the capacity to distinguish between MS-H and its non-temperature sensitive isolates rarely isolated from vaccinated flocks [14].

Microtitration followed by incubation at two different temperatures has been used to determine the temperature-sensitive (*ts*) phenotypes of *M. synoviae* strains/isolates [15]. We have recently developed a technique using a combination of differential growth at two different temperatures with a quantitative real-time PCR (*vlhA* Q-PCR) to determine *ts* phenotype of *M. synoviae* strains [16] however this technique still requires culture of the organism and therefore access to live cloned organism.

We have recently compared partial genome sequences of MS-H, its parent strain 86079/7NS and two *ts* ^–^ MS-H reisolates (MS-H^4^ and MS-H^5^) and found an SNP (G→A) at nucleotide position 367 in MS-H *obg*, an essential gene encoding highly conserved GTP binding protein Obg found in organisms ranging from human to bacteria. Obg is involved in essential cellular processes such as signal transduction, protein synthesis, ribosome biogenesis, DNA replication initiation, chromosomal segregation and progression through cell cycle [17]. A nucleotide change (G→A) at position 367 in MS-H *obg*, causing an alteration of glycine to arginine at position 123 in Obg fold, was predicted to play a role in temperature sensitivity phenotype of MS-H [18]. Analysis of complete *obg* nucleotide sequences from further 19 MS-H reisolates revealed another SNP (C→T) at position 629, causing amino acid change from alanine to valine at position 210 in GTP binding domain, in 4 MS-H reisolates [18]. These SNPs were used in this study to develop a rapid and reliable test, using HRM-curve analysis, to differentiate MS-H from *ts* ^–^ MS-H reisolates and/or *M. synoviae* field strains.

## Materials and Methods

### Ethics statement

Clinical swab samples were taken from palatine cleft, trachea or sinus of specific pathogen free (SPF) chickens, vaccinated with *M. synoviae* vaccine strain MS-H, after euthanasia using intravenous injection of phenobarbitone as per approval of the Melbourne University Animal Ethics Committee (Approval number 0911472.1). Swabs from field commercial chicken flocks were submitted as diagnostic specimens.

### *Mycoplasma* strains and growth media

All mycoplasma strains used in this study are listed in Table 1. A total of 36 *M. synoviae* strains/isolates and 28 clinical swab samples were used in this study. The collection of MS-H reisolates examined in this study (Table 1) is a unique collection prepared in our laboratory through extensive monitoring of the MS-H vaccinated flocks. For 23 out of 36 *M. synoviae* strains/isolates, the *ts* phenotype was determined in a previous study [16]. For MS-H P5, WVU-1853, 94036/5-5a and 94036/6-3a, the *ts* phenotype was determined in this study using microtitration as described before [16]. Other 9 field strains, characterised as different from MS-H based on the analysis of PCR products from *vlhA* gene single-copy conserved region by single strand conformation polymorphism and HRM genotyping [11] (Table 1), were presumed *ts* ^–^. Especially since natural *ts* ^+^ *M. synoviae* strains have not been reported so far, examination of temperature sensitivity of the field strains was not considered in this study. The *ts* ^+^ phenotype is a property of the MS-H vaccine produced by chemical mutagenesis [15],[19]-[21]. Where necessary, *M. synoviae* strains/isolates were grown in mycoplasma broth (MB) containing 10% swine serum and 0.01% (w/v) of nicotinamide adenine dinucleotide (NAD) [22]. *M. gallisepticum* vaccine strain ts-11 was used as control to test the specificity of *obg* PCR primers.

**Table 1:** *Mycoplasma* strains/isolates used in this study.

Mycoplasma species	ID (*ts* phenotype)^a^	Classification	Origin	Specimen type	Reference
M. synoviae	86079/7NS (−)	V,G2	Australia, NSW, parent strain of MS-H vaccine, palatine cleft	Pure culture	[15]
	MS-H (+)	V,G1	Australia, vaccine strain derived from 86079/7NS	Pure culture	[15]
	MS-H P5 (+)	V,G1	MS-H reisolate, Australia (with 5 consecutive in-vivo passages)	Pure culture	This study
	MS-H^4^ (−)	V,G2	MS-H reisolate, Australia	Pure culture	[14]
	MS-H^5^ (−)	V,G2	MS-H reisolate, Australia	Pure culture	[14]
	MS-H^3^ (−)	V,G2	MS-H reisolate, Australia	Pure culture	[14]
	94036/10-5a (−)	V,G2	MS-H reisolate, Australia	Pure culture	[14]
	93198/5-10a (+)	V,G1	MS-H reisolate, Australia	Pure culture	[14]
	93205/1-2a (+)	V,G1	MS-H reisolate, Australia	Pure culture	[14]
	93205/2-9a (−)	V,G2	MS-H reisolate, Australia	Pure culture	[14]
	93198/6-5b (+)	V,G1	MS-H reisolate, Australia	Pure culture	[14]
	93198/4-19a (+)	V,G1	MS-H reisolate, Australia	Pure culture	[14]
	94036/2-2a (+)	V,G1	MS-H reisolate, Australia	Pure culture	[14]
	93198/3-13b (+)	V,G1	MS-H reisolate, Australia	Pure culture	[14]
	93198/3-15a (+)	V,G1	MS-H reisolate, Australia	Pure culture	[14]
	93205/2-13a (−)	V,G2	MS-H reisolate, Australia	Pure culture	[14]
	93205/9-3a (+)	V,G1	MS-H reisolate, Australia	Pure culture	[14]
	93205/8-9c (−)	V,G2	MS-H reisolate, Australia	Pure culture	[14]
	93205/10-13a (−)	V,G2	MS-H reisolate, Australia	Pure culture	[14]
	94036/9-2a (−)	V,G3	MS-H reisolate, Australia	Pure culture	[14]
	93198/6-1a (−)	V,G3	MS-H reisolate, Australia	Pure culture	[14]
	93198/1-24b (−)	V,G3	MS-H reisolate, Australia	Pure culture	[14]
	94036/2-1a (+)	V,G3	MS-H reisolate, Australia	Pure culture	[14]
	94036/5-5a (−)	V,G2	MS-H reisolate, Australia	Pure culture	[14]
	94036/6-3a (−)	V,G2	MS-H reisolate, Australia	Pure culture	[14]
	94036/8-3a (−)	F,G2	Field strain, Australia	Pure culture	[14], [16]
	94041/12a	F,G2	Australia, NSW, field isolate, palatine cleft	Pure culture	[11]
	4GPH3	F,G2	Australia, field isolate, hock joint	Pure culture	[11], [34]
	F10-2AS	F,G2	USA, NC, field strain, airsac	Pure culture	[11]
	K1938	F,G2	USA, AR, field strain	Pure culture	[11]
	K870	F,G2	USA, ME, field strain	Pure culture	[11]
	K1858	F,G2	USA, field strain, trachea	Pure culture	[11]
	YA	F,G2	Source unknown	DNA stock	[11], [35]
	K1968	F,G2	USA, CO, field strain, turkey, joints	Pure culture	[11]
	K1723	F,G2	USA, AR, field strain, trachea	Pure culture	[11]
	WVU-1853 (−)	F,G2	USA, type strain, joints	Pure culture	[11], [36]
	100940-1, -2, -3, -4, -5, -6 and -7 (NA)	S,G2	Australia	Swab samples from non-vaccinated commercial flocks	This study
	100752-A-5T, -B-5T, -C-5T, and -D-5T, 100744-3B	S,G2	Australia	Swab samples from non-vaccinated commercial flocks	This study
	100958-1 to −10 (NA)	S,G2	Australia	Swabs from non-vaccinated commercial flocks	This study
	2774, 2775, 2778, 2781, 2782, 2784 (NA)	S,G1	Australia	Swabs from MS-H vaccinated SPF chickens	This study
M. gallisepticum	ts-11 (+)	NA	Vaccine strain	Pure culture	[37]

a *ts* phenotype was determined in previous studies [16], [33] except MS-H P5, WVU-1853, 94036/5-5a and 94036/6-3a which was determined in this study.

NA, not applicable; V, MS-H vaccine-related strain; F, field strain; S, swab sample; G, genotype, determined by either nucleotide sequencing of *obg* or nested-obg and obg-F3R3 HRMs, based on *obg* SNPs at position 367 and 629.

### DNA extraction

Swabs taken from MS-H vaccinated SPF chickens and non-vaccinated commercial chicken flocks were subjected to DNA extraction. Cell pellet of 500 μl MB culture of *M. synoviae* strains/isolates, harvested by centrifugation at14,000×g for 1 min, or respiratory swabs were placed in 500 μl Qiagen RLT lysis buffer containing 1% of 2-β-mercaptoethanol and incubated at 4°C overnight. After a brief vortex, the swab (where applicable) was removed and 15 μl of Qiaex II matrix (Qiagen, Chadstone, Victoria, Australia) and 300 μl of 70% ethanol added to the lysis buffer. The suspension was mixed and loaded onto a multispin MSK-11 column (Axygen, Union City, California, USA) and placed in a 1.5 ml microfuge tube and centrifuged for 30 sec at 10,000×g with the flow-through discarded. Columns were washed once with 600 μl of RW1 buffer (Qiagen) and twice with 500 μl of RPE buffer (Qiagen) followed by centrifugation for 30 sec at 10,000×g after each wash. The spin column was dried by centrifugation for 90 sec at 14,000×g. Finally, 50 μl of nuclease free water was added to the columns and DNA was eluted after incubation at room temperature for 5 min and centrifugation at 10,000×g for 60 sec. Similar amount of DNA (∼50 ng/μl) was used in all experiments although this was less controllable for clinical specimens submitted as swabs. Extracted DNA was used immediately in PCR or stored at −20°C for future use.

### Oligonucleotide primers

**Figure 1:**
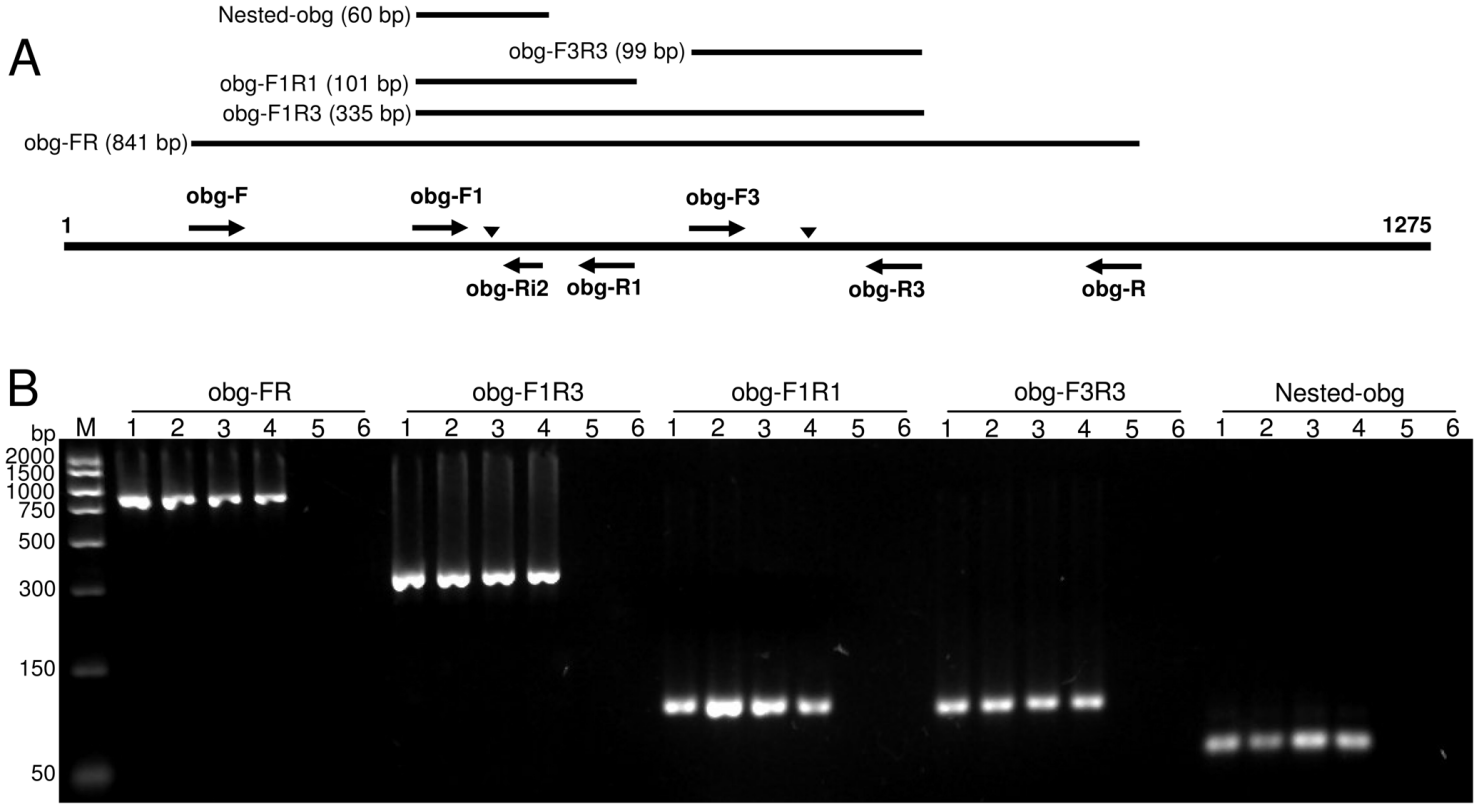
Analysis of different *obg* PCR products by agarose gel electrophoresis. (A) Schematic presentation of *obg* PCRs and the location of primers. Thick and thin lines indicate the extent of full length *obg* and different *obg* PCRs, respectively. Arrows represent primer locations and vertical arrowheads indicate location of *obg* SNPs at positions 367 and 629. (B) Agarose gel electrophoresis of products from *obg* PCRs amplified from MS-H (lane 1), 86079/7NS (lane 2), 94036/1-24b (lane 3), WVU-1853 (lane 4), M. gallisepticum ts-11 (lane 5), and non template control (lane 6). *M, molecular* weight marker (PCR Marker; Sigma, Missouri, USA).

The nucleotide primers used in this study, and their sequences, are listed in Table 2 while their location are shown on Figure 1A. All primers were designed using AmplifX version 1.5.4 and PerlPrimer version 1.1.20 [23]. The primers obg-F1 and obg-R1 were designed to flank *obg* SNP 367. Primers obg-F3 and obg-R3 were designed to flank the *obg* SNP 629. Primers obg-F and obg-R were designed for partial sequencing of the *obg* from *M. synoviae* strains/isolates. Specificity of primers was evaluated using BLAST search against non-redundant nucleotide databases.

**Table 2:** Primers used in this study.

Primers	Sequence (5′ to 3′)	Position^a^	PCR product size (bp)	Application
obg-F	GTTGATAAAGGTGGACCAG	88–106	841	Sequencing
obg-R	TTAGTGCAGATATCTCAATG	928–909		
				
obg-F1	CTTTATTTAGTTGCTAAAGGC	337–357	101	obg-F1R1 HRM
obg-R1	CCGGGCATTCCATTTTCG	437–420		
				
obg-F1	CTTTATTTAGTTGCTAAAGGC	337–357	60	Nested-obg HRM
obg-Ri2	AGAGGTTTTAAATTTATTATTTCC	396–373		
				
obg-F1	CTTTATTTAGTTGCTAAAGGC	337–357	335	obg-F1R3 HRM
obg-R3	CCTTTACCTAGTGATGCG	671–654		
				
obg-F3	TACCTTAGTTCCTCAGTTAGG	573–593	99	obg-F3R3 HRM
obg-R3	CCTTTACCTAGTGATGCG	671–654		

a Nucleotide positions of primers in relation to the DNA sequence of *obg* of MS53, GenBank accession number AE017245.

### *obg* PCRs

Three regions of the *obg*, encompassing both or one of the SNPs detected in MS-H (G→A and C→T at positions 367 and 629, respectively), were targeted by PCR for HRM-curve analysis (Figure 1A). The obg-F1R3 PCR spanned over both SNPs while the obg-F1R1 and obg-F3R3 PCRs spanned over SNP G→A or C→T, respectively. PCR reactions were carried out in iCycler thermal cycler (Bio-Rad, Gladesville, New South Wales, Australia). A 25 μl PCR reaction mixture contained 1 μl each of 25 μM forward and reverse oligonucleotides (0.1 μl each of 25 μM oligonucleotides for obg-F3R3 PCR), 2 μl of 25 mM MgCl_2_, 4 μl of 1.25 mM dNTP mixture, 1 U of GoTaq® DNA polymerase (Promega, Alexandria, New South Wales, Australia), 5 μl of 5×GoTaq® flexi green buffer (Promega), 2 μl of 100 μM SYTO 9 green fluorescent nucleic acid stain (Invitrogen, Mount Waverley, Victoria, Australia), 1 μl of *M. synoviae* genomic DNA (∼50 ng/μl) and 10.8 μl of nuclease free water. PCR reaction conditions for F1R1 PCR included an initial denaturation at 95°C for 2 min, and then 35 cycles of 95°C for 10 sec, 50°C for 20 sec and 72°C for 25 sec. PCR reaction conditions for obg-F1R3 PCR included an initial denaturation at 95°C for 2 min, and then 35 cycles of 95°C for 30 sec, 49°C for 30 sec and 72°C for 30 sec. PCR reaction conditions for obg-F3R3 PCR included an initial denaturation at 95°C for 2 min, and then 35 cycles of 95°C for 30 sec, 58°C for 30 sec and 72°C for 15 sec. In each set of reaction, nuclease free water was used as negative control. All specimens were tested in triplicates.

To amplify a 841-bp region of the *obg* for sequencing purposes, the obg-FR PCR was conducted using oligonucleotide primers obg-F and obg-R. A 50 μl reaction contained 1 μl each of 25 μM oligonucleotide primers, 4 μl of 25 mM MgCl_2_, 8 μl of 1.25 mM dNTP mixture (Promega), 0.3 μl of GoTaq® DNA polymerase (Promega), 10 μl of 5×GoTaq® flexi green buffer (Promega), 22.7 μl of nuclease free water and 3 μl of *M. synoviae* genomic DNA (∼50 ng/μl). PCR conditions included an initial denaturation at 95°C for 2 min then 45 cycles of 95°C for 10 sec, 48°C for 10 sec and 72°C for 60 sec.

All PCR products were analysed by electrophoresis through 1% agarose gels stained with GelRed^™^ (Biotium, Hayward, California, USA) and visualised by UV transillumination.

### High-resolution melting-curve analysis

High-resolution melting-curve analysis was conducted in a Rotor-Gene 6000 thermal cycler (Corbett Life Science, Mortlake, New South Wales, Australia) and signal detected using an excitation wavelength at 470 nm and detection at 510 nm. Melting-curves were generated by increasing the temperature from 60 to 90°C for obg-F1R1, obg-F3R3 and obg-F1R3 PCR products and recording the fluorescence. To optimise melting conditions for maximum differentiation of sequence differences, PCR products were subjected to different ramp speeds of 0.05, 0.1, 0.2, 0.3 and 0.5°C per sec. The HRM-curve analysis was performed using the software Rotor-Gene 1.7.27 and HRM algorithm provided. Conventional melt-curves were generated automatically. To generate normalised HRM-curves, following normalisation regions were applied: 72.5 to 73.0 and 77.5 to 78.0 for obg-F1R1; 70.9 to 71.9 and 79.2 to 80.2 for obg-F3R3 and 74.5 to 76.0 and 80.5 to 82.0 for obg-F1R3. The MS-H profile was set as ‘genotype’ and the average HRM genotype confidence percentages (C%) (value attributed to each strain being compared to the genotype, with a value of 100 indicating an exact match) for replicates were automatically calculated by Rotor-Gene 1.7.27. The C% value attributed to all other strains/isolates indicated similarity of the given strain/isolate to the *ts^+^* MSH. The mean C% of specimen replicates and standard deviations were calculated using Microsoft^™^ Office Excel 2003.

### Nested-obg PCR-HRM

Oligonucleotide primers obg-F1 and obg-Ri2 were used to amplify a 60-bp internal region of *obg* (harbouring the SNP 367) from products generated in obg-F1R1 PCR (Figure 1A). PCR was performed in 25 μl reaction volumes containing 5 μl of 5×GoTaq® flexi green buffer (Promega), 0.1 μl each of 25 μM oligonucleotide primers, 2 μl of 25 mM MgCl_2_, 2 μl of 1.25 mM dNTP mixture (Promega), 0.2 μl of GoTaq® DNA polymerase (Promega), 2 μl of 100 μM SYTO 9 green fluorescent nucleic acid stain (Invitrogen), 2 μl of 0.01× diluted obg-F1R1 PCR product as template and 10.8 μl of nuclease free water. PCR conditions consisted of denaturation at 95°C for 2 min followed by 35 cycles of 95°C for 10 sec, 52°C for 20 sec and 72°C for 10 sec. All reactions were carried out in triplicate. In each experiment, water instead of template was used as negative control and MS-H and parent strain 86079/7NS genomic DNA were used as *ts* ^+^ and *ts* ^–^ controls, respectively. Following PCR, HRM-curve analysis was carried out in Rotor-Gene 6000 thermal cycler (Corbett Life Science Pty Ltd) as described above. Melting curves were generated by increasing the temperature from 66 to 78°C at ramp speeds of 0.1, 0.2, 0.3 and 0.5°C per sec. Normalisation regions of 66.5 to 67.0 and 76.5 to 77.0 and genotype confidence threshold of 85% were applied to characterise unknown *M. synoviae* strains/isolates using MS-H as genotype/reference strain.

### Nucleotide sequencing and sequence analysis

PCR products generated in obg-FR PCR (841-bp) were separated through 1% agarose gels, bands of expected size were excised, purified using Wizard® SV Gel and PCR clean-Up System (Promega) and cloned into pGEM®-T Easy vector (Promega) using instructions provided by the manufacturer. The resultant constructs were propagated in α-select competent cells, silver efficiency (Bioline, Alexandria, New South Wales, Australia) and extracted using PureYield^™^ Plasmid Miniprep (Promega). All purified PCR products or plasmid extracts were subjected to automated sequencing (BigDye Terminator v3.1; Applied Biosystems, Foster City, California, USA) in both directions using primers obg-F and obg-R, or M13 forward and reverse sequencing primers for purified PCR products or cloned PCR products, respectively. Nucleotide sequences were edited using SeqMan^™^ II and EditSeq programs in DNASTAR. Multiple sequences were aligned using computer program ClustalW2. Nucleotide sequence of complete *obg* of 25 strains including MS-H and its related strains, belonging to four different genotypes, has been described in our previous study [18]. Nucleotide sequence of partial *obg* of additional 10 *M. synoviae* strains, including 94041/12a, 4GPH3, F10-2AS, K1938, K870, K1858, YA, K1968, K1723 and WVU-1853, has been submitted to GenBank under accession numbers KF875990 to KF875999.

## Results

### PCR amplification of selected regions of the *obg* from different *M. synoviae* strains/isolates

In order to evaluate the capacity of *obg* PCRs for HRM analysis to differentiate MS-H from *M. synoviae* strains, five sets of oligonucleotide primers, as detailed in Table 2 and Figure 1A, were used to amplify 5 regions of 841, 335, 101, 99 and 60-bp of *obg* from four *M. synoviae* strains/isolates. All strains/isolates generated PCR products of the expected size in all PCRs as confirmed by agarose gel electrophoresis (Figure 1B). No PCR product was detected from *M. gallisepticum* strain ts-11 DNA and no template negative control (Figure 1B) indicating specificity of the *obg* PCRs.

### The obg-F1R3 PCR-HRM curve analysis could not reliably differentiate MS-H from *M. synoviae* strains/isolates tested

The 335-bp obg-F1R3 PCR products from 16 *M. synoviae* strains/isolates including MS-H and its related isolates, and a number of field strains from Australia and the USA were subjected to HRM-curve analysis (Figure S1). Conventional melt-curve analysis of the PCR products using a ramp of 0.3°C/sec showed that all strains generated a single peak at 79.7±0.1°C which were also visually very similar in pattern making it difficult to differentiate MS-H from other strains (Figure S1 and Table S1). Visual examination of the normalised HRM-curves also showed very minor differences between curve profiles of MS-H and other *M. synoviae* strains/isolates (Figure S1). When genotyping was applied to the normalised HRM-curves using MS-H as reference genotype, the C% ± SD for the strains/isolates 93198/1-24b, 94036/5-5a, 4GPH3, K870, WVU-1853 and YA were 78.8±10.0, 83.4±3.8, 71.8±1.6, 87.9±8.8, 89.4±0.5 and 83.2±12.2, respectively. All these strains could be auto-called as ‘variation’ from MS-H when a genotype confidence threshold of 90% was applied. For other strains/isolates, the C% was above 90% (93.7±3.6) and the normalised melt curves were mostly similar to that of MS-H on visual examination. Therefore the obg-F1R3 HRM-curve analysis was not considered as a reliable tool and was not pursued any further in this study for differentiation of MS-H from other strains/isolates.

### The obg-F1R1 PCR-HRM curve analysis differentiated MS-H from all *M. synoviae* field strains/isolates but not from WVU-1853

The 101-bp obg-F1R1 PCR products, spanning over SNP G→A at position 367, from various *M. synoviae* strains/isolates were subjected to HRM-curve analysis. Only a small number of strains/isolates were used in this assay to provide a preliminary evaluation of the assay. Visual examination of conventional melt curves at different ramps revealed that a ramp of 0.3°C/sec generated the most distinct curves and therefore used in the further HRM analysis. The conventional melting-curve analysis showed a single peak for all strains examined. The melting peaks for MS-H vaccine, its *ts* ^+^ reisolates, and the US strain WVU-1853 occurred at 75.8±0.0°C while those for all other strains including 86079/7NS and *ts* ^–^ MS-H reisolates occurred at 76.3±0.1 (Figure S1). Normalised HRM-curve analysis distinctly separated strains into two groups, one for the known *ts* ^+^ and the other for the *ts* ^–^ strains with the exception of the rarely occurring *ts* ^–^ MS-H reisolates with mutation at position 629 (Figure S1). When genotyping with a C% threshold of 90 was applied, two distinct genotypes were auto-called: one included MS-H and *ts* ^+^ MS-H reisolates (mean C%, 97.2±2.6) and the other included *ts* ^–^ strains (mean C%, 6.1±4.7). The MS strain WVU-1853 and the rarely occurring *ts* ^–^ MS-H reisolates had normalised HRM-curves identical to that of MS-H (Figure S1). HRM data from different experiments is shown in Table S2.

### Alignment of partial *obg* nucleotide sequences revealed further SNPs

**Figure 2:**
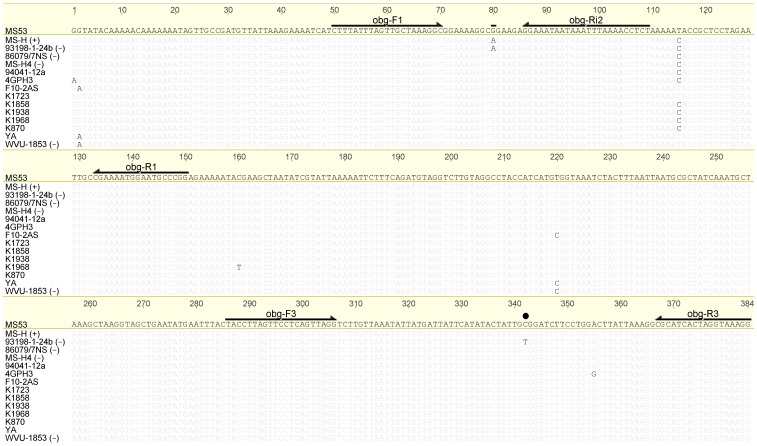
Comparison of partial *obg* nucleotide sequences (corresponding to nt 288-671 of MS53 *obg*; GenBank accession number no. AE017245) from selected *M. synoviae* strains/isolates. Nucleotide differences are highlighted keeping MS53 as reference. Location of primers used in *obg* PCRs as well as SNP G367A discovered in MS-H genome are highlighted with arrows and bar above the sequence, respectively. Location of SNP C629T, observed only in 93198/6-1a, 93198/1-24b, 94036/9-2a and 94036/2-1a [18], is also highlighted with a dot.

Alignment of partial nucleotide sequence of *obg* from MS-H, MS-H reisolates (both *ts* ^+^ and *ts* ^–^) and field *M. synoviae* strains/isolates revealed further nucleotide variations in *obg* (Figure 2), especially in the region targeted in obg-F1R1 PCR. *M. synoviae* strains F10-2AS, K1723, YA and WVU-1853 had C→T variation at position 402 while 94041/12a had C→A variation at position 434. Therefore, a further oligonucleotide primer (obg-Ri2) was designed to allow targeting of the region spanning over the SNP G→A at position 367 and avoiding other polymorphic sites found in *obg*. Nucleotide sequence alignment of *obg* regions targeted in nested-obg and obg-F3R3 PCR-HRM, for all *M. synoviae* strains/isolates used in this study except field isolate 94036/8-3a, is shown in Figure S2 and S3, respectively. For reasons unknown to the authors, several attempts at sequencing the obg-FR PCR product for 94036/8-3a were failed. Similarly, attempts at sequencing the *vlhA* region of 94036/8-3a were unsuccessful in our previous study [16].

### Nested-obg PCR-HRM curve analysis differentiated MS-H from most of the *M. synoviae* field strains/isolates and MS-H reisolates

HRM-curve analysis of the nested-obg PCR product (60-bp in size) at a ramp rate of 0.3°C/sec revealed a single peak of 72.3±0.1°C for MS-H and of 73±0.0°C for 86079/7NS. Visual examination of the conventional and normalised HRM-curves revealed that all known *ts* ^+^ MS-H reisolates generated HRM-curves similar to those for MS-H while the *ts* ^–^ *M. synoviae* strains/isolates and *ts* ^–^ MS-H reisolates (except 93198/1-24b, 94036/9-2a and 93198/6-1a) had HRM-curves similar to those for 86079/7NS. After applying genotyping to the normalised curves using a C% threshold of 84, two distinct genotypes were auto-called: the MS-H type with a mean C% of 96.9±3.4, and variants with a mean C% of 8.8±11.2. MS-H reisolates (with a *ts* ^–^ phenotype) that previously could not be differentiated from MS-H, due to identical *vlhA* region, were distinguishable from MS-H in the nested-obg PCR-HRM (Table 3).

**Table 3:** Melting points and genotype confidence percentages (C%) generated in nested-obg HRM from different *M. synoviae* strains/isolates.

Strains/isolates	Nested-obg		
	Melting points (°C) (Mean ± SD)^a^	HRM-curve genotype	C% (Mean ± SD)^a^
MS-H	72.3±0.1	MS-H	99.3±0.5
86079/7NS	73.0±0.0	Variation	5.1±2.2
94036/10-5a	73.0±0.0	Variation	2.9±0.3
93198/5-10a	72.3±0.0	MS-H	97.3±2.6
b93198/6-1a	72.3±0.1	MS-H	97.5±3.9
b93198/1-24b	72.3±0.1	MS-H	97.9±2.4
b94036/9-2a	72.3±0.1	MS-H	98.8±1.7
b94036/2-1a	72.3±0.1	MS-H	98.7±0.8
MS-H^4^	73.0±0.1	Variation	3.9±1.9
93205/1-2a	72.3±0.1	MS-H	97.5±2.5
93205/2-9a	73.0±0.0	Variation	4.7±1.7
93198/6-5b	72.4±0.1	MS-H	97.2±3.3
94036/5-5a	73.0±0.1	Variation	28.6±10.4
94036/6-3a	73.0±0.0	Variation	7.3±2.4
93198/4-19a	72.3±0.0	MS-H	99.0±1.2
94036/2-2a	72.4±0.1	MS-H	87.1±3.4
93198/3-13b	72.4±0.1	MS-H	94.4±3.9
93198/3-15a	72.4±0.0	MS-H	98.7±0.8
94041/12a	73.1±0.0	Variation	6.5±0.9
94036/8-3a	72.5±0.0	Variation	45.8±0.9
4GPH3	73.1±0.1	Variation	6.9±3.8
F10-2AS	73.1±0.1	Variation	6.4±1.6
K1938	73.1±0.0	Variation	4.1±0.7
K870	73.1±0.1	Variation	4.9±2.7
K1858	73.1±0.1	Variation	4.3 ± 2.5
YA	73.1±0.0	Variation	4.6±1.8
K1968	73.0±0.1	Variation	5.9±2.7
K1723	73.1±0.0	Variation	4.9±0.7
WVU-1853	73.0±0.0	Variation	3.5±0.3

a Melting points and C% values are from one HRM experiment for each strain using each sample DNA tested in triplicate. HRM data for all strains/isolates and swab samples, from different experiments, is shown in Table S3.

b These strains are rarely occurring *ts* ^–^ MS-H reisolates [18] and can be discriminated from MS-H using obg-F3R3 HRM.

**Figure 3:**
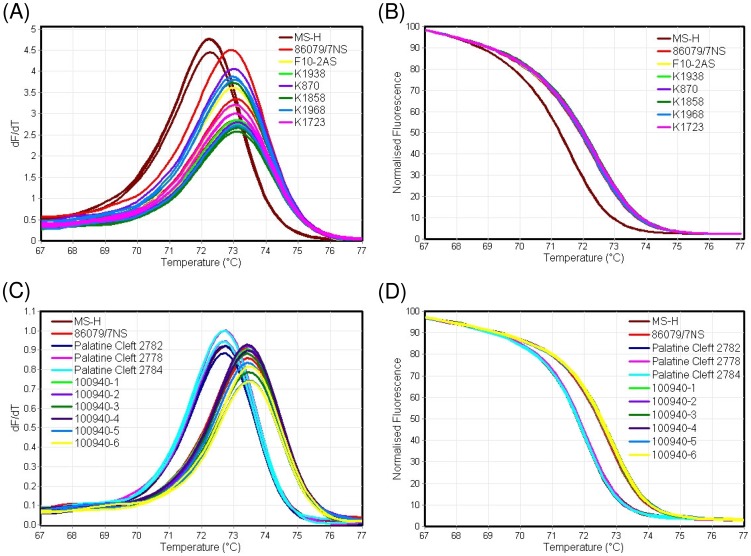
High-resolution melting-curve analysis of *M. synoviae* strains/isolates using nested-obg PCR products. (A) Conventional and (B) normalised melt-curves of DNA extracted from pure cultures of *M. synoviae* field strains/isolates indicated 86079/7NS-like genotype, and therefore characterised as *ts* ^–^. (C) Conventional and (D) normalised melt-curves of DNA extracted from swabs taken from MS-H vaccinated SPF chickens (palatine cleft 2782, 2778 and 2784) and non-vaccinated commercial chicken flocks (100940-1, -2, -3, -4, -5 and -6). Samples from vaccinated chickens were genotyped as MS-H-like while from non-vaccinated as 86079/7NS-like.

Nested-obg HRM melting points for MS-H, 86079/7NS and other strains grouped with either of them exhibited minor variation in melting temperature on different days using different DNA extractions as templates but the melting point differences between MS-H and 86079/7NS remained ≥0.7°C (Table S3). Normalised HRM-curves, in all instances, correctly genotyped all strains/isolates either with MS-H or 86079/7NS. Furthermore, all tested US strains (F10-2AS, K1723, K1858, K1938, K1968, K870, YA and WVU-1853) were autocalled as variant from MS-H genotype and produced melting-curves (73.1±0.0°C) and C% (5.1±1.8) identical to 86079/7NS, and therefore, characterised as *ts* ^–^ (Figure 3A and B and Table 3). HRM data for all (36) *M. synoviae* strains/isolates, used in this study, is shown in Table S3.

### Nested-obg PCR-HRM curve analysis successfully applied for direct examination of clinical specimens

The nested-obg PCR-HRM was first optimised using DNA extracted from pure cultures of *M. synoviae* strains/isolates as described above, and then extended to clinical swab specimens taken from sinus, palatine cleft or trachea of SPF and field chickens inoculated intra-ocularly with MS-H. Swabs from palatine cleft and trachea of non-vaccinated commercial chicken flocks were used as negative control. All swabs from MS-H vaccinated SPF chickens produced single melting peak at 72.0±0.0°C while swab samples from non-vaccinated commercial field chicken flocks generated peak at 72.8±0.0°C. Melting peaks for MS-H and 86079/7NS as *ts* ^+^ and *ts* ^–^ controls were 72.0±0.0°C and 72.8±0.0°C, respectively, thus visual examination of the melting-curves could clearly differentiate the two different melting profiles (Figure 3C). Application of genotyping on normalised curves (using MS-H as reference), distinctively classified the specimens either as MS-H (mean C% of 99.3±0.6) or variation (4.1±1.3) (Figure 3D). Therefore there was an approximate gap of 95% in C% for these two groups. HRM data for all swab samples tested in this study is shown in Table S3.

### The obg-F3R3 PCR-HRM curve analysis differentiated MS-H from rare variants of MS-H reisolates and field strains but not from its parent strain 86079/7NS

**Figure 4:**
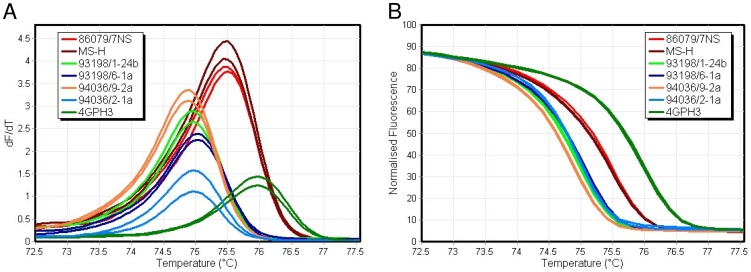
High-resolution melting-curve analysis of *M. synoviae* strains/isolates using obg-F3R3 PCR products. (A) Conventional and (B) normalised melt-curves distinguished MS-H from rarely occurring *ts* ^–^ (93198/1-24b, 93198/6-1a, 94036/9-2a) and *ts* ^+^ (94036/2-1a) MS-H reisolates and field strains (e.g., 4GPH3).

HRM-curve analysis of 99-bp obg-F3R3 PCR products (encompassing C→T SNP at position 629 in 4 MS-H variants examined), at a ramp of 0.2°C/sec, revealed a single peak at 75.5±0.0°C for MS-H and 86079/7NS and at 75.0±0.1°C for MS-H reisolates 93198/1-24b, 94036/9-2a, 93198/6-1a and 94036/2-1a. The Australian strain 4GPH3 with one base replacement at position 642 of the *obg*, generated a distinguishable (from that of MS-H) single peak at 76.0±0.0°C. Normalised HRM-curves discriminated MS-H from strains/isolates 93198/1-24b, 94036/9-2a, 93198/6-1a and 94036/2-1a, and the field strain 4GPH3, but not from 86079/7NS. When MS-H was selected as genotype, the mean C% of 93198/1-24b, 94036/9-2a, 93198/6-1a and 94036/2-1a was calculated as 8.0±4.8 (Table 4 and Figure 4). For field strain 4GPH3 the mean C% was 1.4±0.2.

**Table 4:** Melting points and genotype confidence percentages (C%) generated in obg-F3R3 HRM from different *M. synoviae* strains/isolates.

Strains/isolates	obg-F3R3		
	Melting points (°C)(Mean ± SD)^a^	HRM-curve genotype	C% (Mean ± SD)^a^
MS-H	75.5±0.0	MS-H	99.9±0.1
86079/7NS	75.5±0.0	MS-H	98.8±1.7
b93198/6-1a	75.0±0.0	Variation	12.9±0.9
b93198/1-24b	75.0±0.0	Variation	7.6±3.1
b94036/9-2a	74.9±0.0	Variation	1.6±0.2
b94036/2-1a	75.0±0.0	Variation	10.7±1.6
4GPH3	76.0±0.0	Variation	1.4±0.2

a Melting points and C% values are from one HRM experiment for each strain using each sample DNA tested in triplicate.

b These strains are rarely occurring *ts* ^–^ MS-H reisolates [18] and can only be discriminated from MS-H using obg-F3R3 HRM.

Thus, coupling of nested-obg with obg-F3R3 PCR-HRM had 100% accuracy in differentiation of MS-H from all field strains and *ts* ^–^ MS-H reisolates. Also a high accuracy (97.2%) was achieved in predicting the *ts* phenotype of *M. synoviae* strains/isolates (Table 5). Irrespective of the (unknown) prevalence of strains with identical nucleotide at position 629 to that of MS-H (e.g., 86079/7NS), the presence of isolates with SNP at position 629 reflects that obg-F3R3 PCR is more useful when combined with the nested obg-PCR.

**Table 5:** *M. synoviae obg* SNPs-based genotyping scheme and its association with ts phenotype.

Genotypes	SNPs in *obg*		No. of isolates/*ts* phenotype^a^ (n = 36)	PCR-HRM for MS-H genotyping
	367	629		
G1 (MS-H)	A	C	10/+	Nested-obg
G2	G	C	22/−	Nested-obg
G3	A	T	3/−	Nested-obg combined with obg-F3R3
G3	A	T	1/+	Nested-obg combined with obg-F3R3

a *ts* phenotype was determined by conventional microtitration method [16].

## Discussion

SNPs are the most common type of genetic variation and have been used for species/strain identification of various bacterial pathogens [24]–[29]. A large number of methods have been utilised for rapid identification of SNPs. These include hybridisation-based methods (molecular beacons, SNP microarrays), enzyme-based methods (restriction fragment length polymorphism, flap endonuclease, primer extension, 5′-nuclease, oligonucleotide ligase assay) and post-amplification methods based on physical properties of the DNA (single strand conformation polymorphism, temperature gradient gel electrophoresis, high resolution melting analysis) [30]. Among these, HRM is thought to be rapid and at the same time most economical where a small number of specimens are to be genotyped [31], [32].

In this study, a set of SNPs detected by comparative genomic sequence analysis of *M. synoviae* strains of MS-H lineage were analysed. A selection of MS-H strain specific SNPs was confirmed by Sanger sequencing. SNPs in *obg* associated with change in temperature sensitivity [18] were primarily targeted to enable differentiation of MS-H strain from *ts* ^–^ MS-H revertants as well as from *ts* ^–^ field strains. Initially a 335-bp obg-F1R3 PCR-HRM was developed but melt curves generated did not provide clear differentiation of MS-H from other strains/isolates. This failure may be related to the small number of sequence variations over a relatively large amplicon and/or balancing effect of the mutations at positions 367 and 629. Previous studies in our group have shown that one nucleotide difference in approximately 400 bp target sequence might be sufficient for differentiation of highly similar sequences [11] but differentiation power of a HRM system may also be influenced by the location of the SNP as well as structure of the DNA surrounding it. In order to develop a more reliable assay for the detection of *obg* SNPs, an alternative PCR (obg-F1R1 PCR) was developed that targeted a smaller (101-bp) region of *obg*, encompassing the *obg* SNP G367A. The obg-F1R1 PCR could differentiate MS-H from *ts* ^–^ MS-H reisolates and *ts* ^–^ field strains/isolates. However, four field strains (F10-2AS, K1723, WVU-1853 and YA) generated HRM-curves identical to that of MS-H. Despite this limitation, obg-F1R1 PCR-HRM was still considered useful when combined with other strain identification techniques such as *vlhA* gene sequence analysis. To further differentiate MS-H from its closely related isolates, a nested-obg PCR-HRM, targeting a 60-bp region of the *obg* encompassing the SNP G367A was developed. With the exception of 93198/1-24b, 93198/6-1a and 94036/9-2a, the nested-obg PCR-HRM was able to differentiate MS-H from all other field strains/isolates and *ts* ^–^ MS-H reisolates.

The potential of nested-obg PCR-HRM was initially evaluated using pure *M. synoviae* cultures available in our laboratory. In order to evaluate the potential of the nested-obg PCR-HRM directly on clinical specimens, two sets of clinical swabs, one from MS-H vaccinated SPF birds and the other from commercial chicken flocks suspected (by serological monitoring) of *M. synoviae* infection were tested. The swabs from MS-H vaccinated SPF birds generated HRM-curves identical to that of MS-H culture while swab samples from infected field birds produced clearly different pattern, identical to that generated by 86079/7NS, indicating infection by a field strain at the time of sampling. This demonstrated the potential of the nested-obg PCR-HRM to rapidly determine whether a flock is infected with a field *M. synoviae* strain or it harbours MS-H vaccine strain. Evaluation of the full potential of this assay for direct examination of clinical specimens should include determination of its sensitivity and specificity although it should be noted that the primary use of this assay in our laboratory has been confined to examination of pure (cloned) *M. synoviae* cultures.

The nested-obg HRM also discriminated *ts* ^+^ MS-H from *ts* ^–^ field strains and *ts* ^–^ MS-H reisolates. Out of total 36 *M. synoviae* strains/isolates, 33 were typed according to their *ts* phenotype. The *ts* phenotype of three MS-H reisolates (93198/1-24b, 93198/6-1a and 94036/9-2a) was not determined in accordance with their temperature sensitivity phenotype (genotyped as ‘MS-H’ by nested-obg PCR-HRM but exhibited *ts* ^–^ phenotype). Complete *obg* sequence of these three strains and one MS-H reisolate 94036/2-1a (*ts* ^+^) revealed a secondary mutation (C→T) at position 629. Therefore, an alternative PCR-HRM (obg-F3R3 PCR-HRM), encompassing SNP at position 629 was developed to discriminate these rare variants from all other *M. synoviae* strains/isolates. It is recommended that where an unknown *M. synoviae* strain/isolate genotyped as MS-H by the nested-obg HRM, the obg-F3R3 HRM should be used to determine its *ts* phenotype and identity. Thus, a combination of nested-obg and obg-F3R3 HRM-curve analysis not only differentiates MS-H from field *M. synoviae* strains and from *ts* ^–^ MS-H reisolates, but also exhibited high accuracy (97.2%, 35/36) in predicting the *ts* phenotype of any unknown *M. synoviae* strain/isolate. The exception was the rare isolate 94036/2-1a (*ts* ^+^) with *obg* mutations at position 367 and 629. The influence of this second mutation on *ts* phenotype of *M. synoviae* has been discussed in our previous report [18].

Temperature-sensitive bacterial mutants have been produced only in laboratories, mostly by N-methyl-N-nitro-nitrosoguanidine (NTG) [15], [19]-[21]. Such *ts* ^+^ mutants are expected to sustain more than one *ts* mutation contributing to overall *ts* phenotype and accounting for the genetic stability of temperature-sensitive mutants [20]. Therefore *obg* SNPs based assays developed in this study may not be ideal for *ts* phenotyping of other organisms although due to their consistency for the MS-H and its reisolates, were found highly useful for *M. synoviae* genotyping purposes.

Wild strain mutation causing *ts* ^+^ (MS-H-like phenotype) has never been reported to the best of our knowledge. However reversion from a *ts* ^+^ to *ts* ^–^ phenotype is expectable under favourable selective pressure [15], [33]. The back mutation rate of the MS-H vaccine strain from a *ts* ^+^ phenotype to *ts* ^–^ phenotype was found in the order of 10^-4^. No study on the virulence and transmissibility of *ts* ^+^ MS-H reisolates have been conducted although a previous study in our laboratory demonstrated that *ts* ^–^ MS-H reisolates did not have the characteristics, including virulence potential, of the vaccine parent strain [33] and that factors other than *ts* phenotype may be involved in loss of virulence of MS-H. Nevertheless the recovery of *ts* ^–^ strain/isolate from healthy vaccinated flocks prompted the current study to establish the true identity of the isolate.

The combination of nested-obg and obg-F3R3 PCR-HRM is relatively rapid and can be completed in one day after DNA extraction. High discriminating power of this genotyping system, with an added advantage of predicting the *ts* phenotype, makes it an ideal assay that can be routinely used in veterinary diagnostic laboratories involved in *M. synoviae* genotyping especially in countries where MS-H is routinely used in commercial poultry.

## Supporting Information

Figure S1 High-resolution melting-curve analysis of *M. synoviae* strains/isolates using obg-F1R3 and obg-F1R1 PCR products. Using obg-F1R3 HRM, conventional (A) and normalised melt-curves (B) of *M. synoviae* strains were almost identical and thus could not differentiate MS-H from field strains or *ts*– MS-H reisolates. Using obg-F1R1 HRM, conventional (C) and normalised melt-curves (D) of MS-H were distinguishable from all other strains except *M. synoviae* reference strain WVU-1853 and rarely occurring *ts*– MS-H reisolates with *obg* mutation at position 629.
(TIF)

Figure S2 Partial *obg* nucleotide sequence alignment for 35 *M. synoviae* strains/isolates encompassing region harbouring SNP 367.
(PNG)

Figure S3 Partial *obg* nucleotide sequence alignment for 35 *M. synoviae* strains/isolates encompassing region harbouring SNP 629.
(PNG)

Table S1 Melting points and genotype confidence percentages (C%) in obg-F1R3 HRM for different *M. synoviae* strains/isolates.
(DOC)

Table S2 Melting points and genotype confidence percentages (C%) in obg-F1R1 HRM for *M. synoviae* strains/isolates from different experiments.
(XLS)

Table S3 Details of melting points and genotype confidence percentages (C%) in nested-obg HRM for *M. synoviae* strains/isolates from different experiments.
(XLS)
