# Supplementary figures and images for: High-Resolution Melting-Curve Analysis of obg Gene to Differentiate the Temperature-Sensitive Mycoplasma synoviae Vaccine Strain MS-H from Non-Temperature-Sensitive Strains
Source: PLoS One. 2014 Mar 18;9(3):e92215. doi: 10.1371/journal.pone.0092215 (PMC3958494; doi:10.1371/journal.pone.0092215)

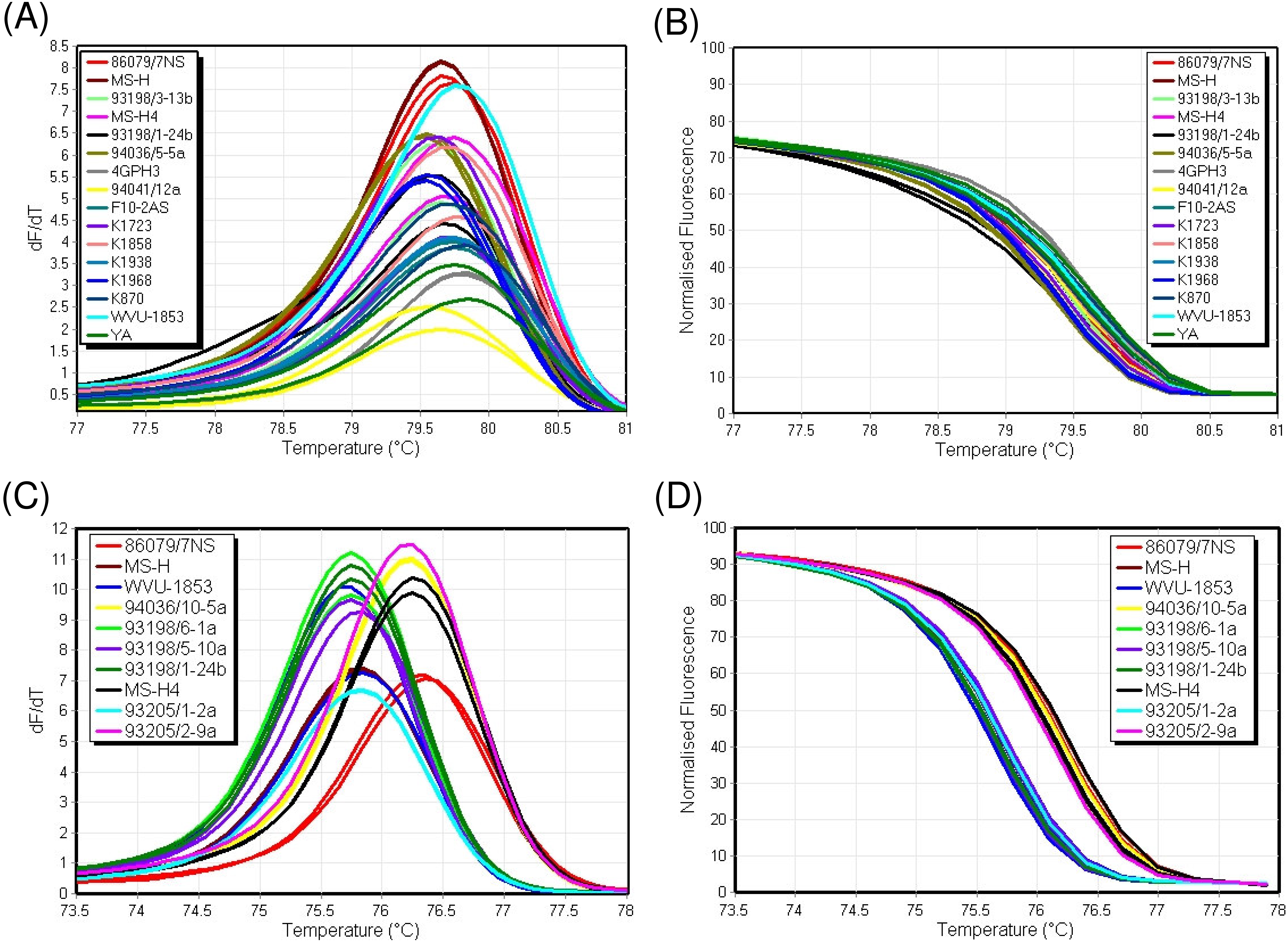

Supplement: Figure S1 — High-resolution melting-curve analysis of M. synoviae strains/isolates using obg-F1R3 and obg-F1R1 PCR products. Using obg-F1R3 HRM, conventional (A) and normalised melt-curves (B) of M. synoviae strains were almost identical and thus could not differentiate MS-H from field strains or ts– MS-H reisolates. Using obg-F1R1 HRM, conventional (C) and normalised melt-curves (D) of MS-H were distinguishable from all other strains except M. synoviae reference strain WVU-1853 and rarely occurring ts– MS-H reisolates with obg mutation at position 629. (TIF) [file pone.0092215.s001.tif]

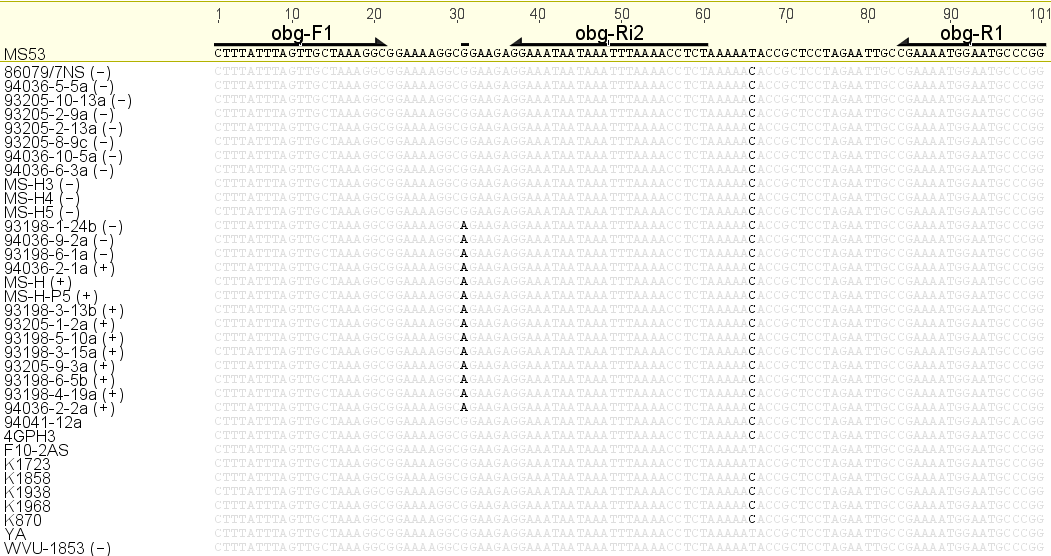

Supplement: Figure S2 — Partial obg nucleotide sequence alignment for 35 M. synoviae strains/isolates encompassing region harbouring SNP 367. (PNG) [file pone.0092215.s002.png]

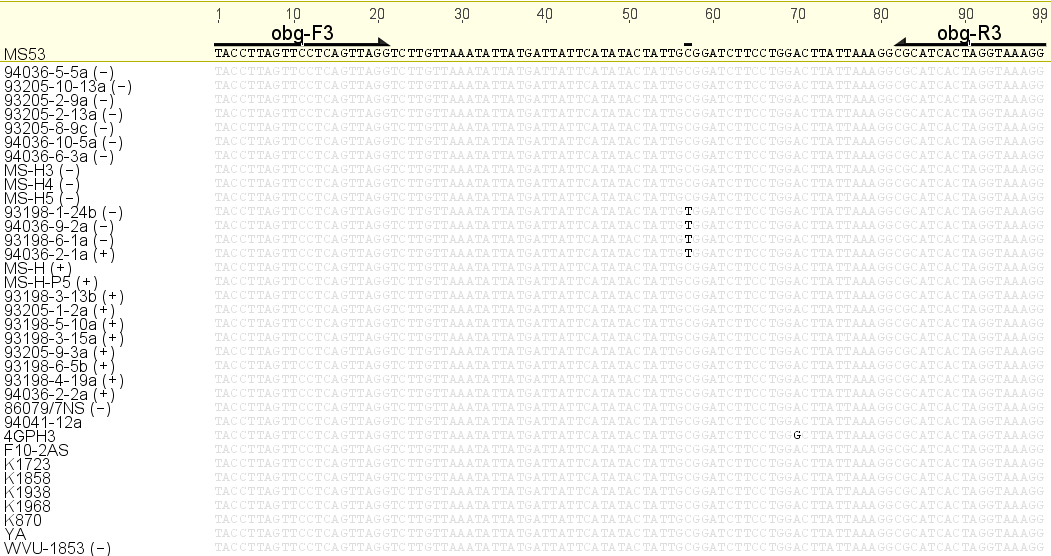

Supplement: Figure S3 — Partial obg nucleotide sequence alignment for 35 M. synoviae strains/isolates encompassing region harbouring SNP 629. (PNG) [file pone.0092215.s003.png]
