# Supplementary material for: High-Resolution Melting-Curve Analysis of obg Gene to Differentiate the Temperature-Sensitive Mycoplasma synoviae Vaccine Strain MS-H from Non-Temperature-Sensitive Strains
Source: PLoS One. 2014 Mar 18;9(3):e92215. doi: 10.1371/journal.pone.0092215 (PMC3958494; doi:10.1371/journal.pone.0092215)
Supplement: Table S1 — Melting points and genotype confidence percentages (C%) in obg-F1R3 HRM for different M. synoviae strains/isolates. (DOC) [file pone.0092215.s004.doc]

Table S1. Melting points and genotype confidence percentages (C%) in obg-F1R3 HRM for different *M. synoviae* strains/isolates

| Strains/isolates | obg-F1R3 | | |
| --- | --- | --- | --- |
|  | Melting points (°C) (Mean ± SD) | HRM genotype | C% (Mean ± SD) |
| 86079/7NS | 79.7 ± 0.0 | MS-H | 97.6 ± 2.5 |
| MS-H | 79.7 ± 0.0 | MS-H | 100 ± 0.0 |
| 93198/3-13b | 79.6 ± 0.0 | MS-H | 98.2 ± 2.4 |
| MS-H^4^ | 79.7 ± 0.0 | MS-H | 93.2 ± 3.3 |
| ^a^93198/1-24b | 79.6 ± 0.0 | Variation | 78.8 ± 10.0 |
| 94036/5-5a | 79.5 ± 0.0 | Variation | 83.4 ± 3.8 |
| 4GPH3 | 79.8 ± 0.0 | Variation | 71.8 ± 1.6 |
| 94041/12a | 79.6 ± 0.1 | MS-H | 95.2 ± 1.7 |
| F10-2AS | 79.7 ± 0.0 | MS-H | 91.5 ± 1.0 |
| K1723 | 79.7 ± 0.0 | MS-H | 97.2 ± 0.7 |
| K1858 | 79.7 ± 0.1 | MS-H | 90.9 ± 6.8 |
| K1938 | 79.7 ± 0.0 | MS-H | 92.7 ± 0.6 |
| K1968 | 79.5 ± 0.0 | MS-H | 90.7 ± 2.6 |
| K870 | 79.7 ± 0.1 | Variation | 87.9 ± 8.8 |
| WVU-1853 | 79.8 ± 0.0 | Variation | 89.4 ± 0.5 |
| YA | 79.8 ± 0.1 | Variation | 83.2 ± 12.2 |

^a^ rarely occurring *ts*^–^ MS-H reisolate [18]
